# Individual expression features of *GPX2, NQO1* and *SQSTM1* transcript variants induced by hydrogen peroxide treatment in HeLa cells

**DOI:** 10.1590/1678-4685-GMB-2016-0005

**Published:** 2017-05-29

**Authors:** Anna A. Belanova, Dmitry S. Smirnov, Maxim S. Makarenko, Mariya M. Belousova, Elena V. Mashkina, Anzhela A. Aleksandrova, Alexander V. Soldatov, Peter V. Zolotukhin

**Affiliations:** 1 Evolution Corporate Group, Cell Physiology Laboratory, Southern Federal University, Rostov-on-Don, Russia; 2 Academy of Biology and Biotechnology, Southern Federal University, Rostov-on-Don, Russia; 3 Department of English Language for Natural Sciences Faculties, Southern Federal University, Rostov-on-Don, Russia; 4 Department of Nanosystems Physics and Spectroscopy, Southern Federal University, Rostov-on-Don, Russia

**Keywords:** NFE2L2/AP-1 pathway, interactomics, transcript variants expression control

## Abstract

Pathway activity assessment-based approaches are becoming highly influential in various fields of biology and medicine. However, these approaches mostly rely on analysis of mRNA expression, and total mRNA from a given locus is measured in the majority of cases. Notably, a significant portion of protein-coding genes produces more than one transcript. This biological fact is responsible for significant noise when changes in total mRNA transcription of a single gene are analyzed. The NFE2L2/AP-1 pathway is an attractive target for biomedical applications. To date, there is a lack of data regarding the agreement in expression of even classical target genes of this pathway. In the present paper we analyzed whether transcript variants of *GPX2, NQO1* and *SQSTM1* were characterized by individual features of expression when HeLa cells were exposed to pro-oxidative stimulation with hydrogen peroxide. We found that all the transcripts (10 in total) appeared to be significantly individually regulated under the conditions tested. We conclude that individual transcripts, rather than total mRNA, are best markers of pathway activation. We also discuss here some biological roles of individual transcript regulation.

## Introduction

Pathway activity assessment-based approaches are becoming more and more influential in various and diverse fields of biology and medicine: in environmental monitoring (Shukla *et al.*, 2012), general (Subramaniam and Ellis, 2011; Chakraborty *et al.*, 2014; Shkurat *et al.*, 2014) and personalized (Wu *et al.*, 2010) pharmacology, pathophysiology (Zolotukhin *et al.*, 2014a), diagnostics (Zolotukhin *et al.*, 2014b; Yao *et al.*, 2015), and patient follow-up (Sibhatu *et al.*, 2008; Shimizu *et al.*, 2015). Most of these approaches rely on RNA expression analysis, because measuring transcription rate is the most representative means to assess pathway activation known to date. However, individual control of the transcript variant expression is ignored in most cases.

Yet, a pathway may control transcripts' fate individually on several levels. Firstly, transcription factors of the pathway can directly induce individual transcripts (Jyrkkänen *et al.*, 2011). Secondly, transcription factors, being central to some pathways, can attract and regulate splicing machinery themselves (Pan *et al.*, 2003). Thirdly, other pathway components can easily regulate splicing machinery together with promoting target gene transcription (Yadav *et al.*, 2014). Fourthly, cellular pathways have all capabilities to individually control degradation or long-term storage of mature mRNA variants of a single gene (Shim and Karin, 2002; Rattenbacher and Bohjanen, 2012; Kurinna and Werner, 2015).

Thus, individual transcripts, rather than total mRNA read from a single locus appear to be far more valuable and adequate for purposes of pathway activity assessment. Yet, individual transcript expression-based studies are rare due to technical and interpretative difficulties (Arseneau *et al.*, 2009; Boudreau *et al.*, 2011).

One of highly informative pathways used today in all the above mentioned biology and medicine fields is the NFE2L2/AP-1 pathway (sometimes subdivided into NFE2L2 (Nrf2) and AP-1 pathways in the literature). Despite attracting much attention, this pathway is poorly studied in terms of regulation of expression of transcripts of target genes of this pathway. Our laboratory previously contributed to solving the problem by showing that *TXN* transcript variants are differentially regulated upon NFE2L2/AP-1 activation (Dovzhik *et al.*, 2014). Still, not much is done for other genes, even those frequently used as pathway activation markers in numerous studies (Marrot *et al.*, 2008; MacLeod *et al.*, 2009; Zhang *et al.*, 2010; Subramaniam and Ellis, 2011; Wang *et al.*, 2014). At the same time, pathway activation would be much easier to detect and, which is even more important, with greater specificity, if individual transcripts were used as pathway activation markers. The basis for this consideration is the fact that when, for example, one of the three transcripts of a given locus may be induced, the second one suppressed and the third one unchanged, total mRNA from this locus suggests expression to be unchanged and the pathway to be not activated at all, which is obviously a false negative result.

In addition to the practical implication, differential regulation of transcript variants is of great biological significance. Transcript variants fold differently and thus interact differently with RNA-transporting and RNA-processing proteins. This difference in interactions makes it possible for the cell to tightly regulate the speed of response towards stimuli: transcript variants differ quantitatively and qualitatively in storage/retention properties, rate of degradation and speed of translation. This biological matter is also rarely addressed in studies.

Considering these issues, we decided to test whether three important NFE2L2/AP-1 pathway targets with with more than one mRNA form could indicate individual transcript regulation. These genes were *GPX2, NQO1* and *SQSTM1*.

GPX2, a cytosolic enzyme reducing peroxides using glutathione as the substrate, is one of seven known proteins of the human family of glutathione peroxidases (GPX8 is still considered a probable glutathione peroxidase). The gene coding for this protein is peculiar: its basal and inducible expression is mostly and almost directly regulated by NFE2L2 (Singh *et al.*, 2006; Sykiotis and Bohmann, 2010), and its ARE is well-studied (Singh *et al.*, 2006). In contrast, the *NQO1* gene, one of the first known NFE2L2 targets, has multiple transcription factors controlling it, although in some cells, *NQO1* is also controlled mostly by NFE2L2 (Marrot *et al.*, 2008). The *NQO1* NFE2L2 binding site is also thoroughly described: it is characterized by two core ARE sequences with one embedded TRE sequence (Venugopal and Jaiswal, 1996; Kim *et al.*, 2011). *NQO1* codes for a multi-functional enzyme scavenging superoxide anion (Siegel *et al.*, 2004; Dinkova-Kostova and Talalay, 2010), reducing quinones and thus blocking redox-cycling (Jaiswal, 2000), and protecting the nucleus from pro-oxidants (Winski *et al.*, 2002) as well as associating with mitotic spindle (Siegel *et al.*, 2012). NQO1 is even known to stabilize p53 (officially known as TP53) (Dinkova-Kostova and Talalay, 2010). The autophagosomal adaptor protein SQSTM1 (also known as p62) is capable of activating the NFE2L2 sub-pathway without oxidative modification of the KEAP1 protein (Copple *et al.*, 2010; Bui and Shin, 2011). The mode of antioxidant action of this factor is in line with its primary function, as it merely targets KEAP1 for autophagosomal degradation (Copple *et al.*, 2010; Bui and Shin, 2011). Interestingly, in a murine model, Sqstm1-dependent activation of Nrf2 was responsible for approximately 50% of basal expression of classical Nfe2l2 targets: *Nqo1, Gclc,* and *Hmox1* (Copple *et al.*, 2010). The ARE of *SQSTM1* was proven to be functional, and this gene is another NFE2L2 target (Jain *et al.*, 2010).

The aim of the study was to assess whether *GPX2, NQO1* and *SQSTM1* transcript variants were regulated individually when cells were exposed to hydrogen peroxide treatment, a classical NFE2L2/AP-1 activation stimulus.

## Materials and Methods

The study was carried out in 2015 at the Southern Federal University Academy of Biology and Biotechnology Shared Equipment Centre.

### Cell culture, hydrogen peroxide treatment and viability assay

In this study, the HeLa cell line was used as an experimental model. The cells were kindly provided by Southern Scientific Center of the Russian Academy of Science and validated by cytogenetic (G-staining) and molecular genetic analyses. The cells were grown in T25 flasks, 24- and 96-well plates (SPL Lifesciences, South Korea) in GlutaMax DMEM medium (Thermo Fisher Scientific, USA) supplemented with 10% of fetal bovine serum (GE Healthcare, UK) and 0.05 μg/ml of gentamicin (Biokhimik JSC, Russia). The cells were kept at 37 °C and 5% CO_2_, with passive humidification in the Sanyo MCO-18AC incubator (Panasonic, Japan). Cell growth was controlled using the Premiere MIS-9000 inverted microscope (C&A, China).

As the NFE2L2/AP-1 pathway is activated by pro-oxidants, we used hydrogen peroxide as a convenient treatment substance. It is a physiological compound and its injection into the medium does not introduce any additional metabolites that could affect the performance of the classical variant of the pathway by activating various upstream kinases and adjacent pathways further indirectly affecting the NFE2L2/AP-1 pathway. Hydrogen peroxide stock solution (ProChem LLC, Russia) concentration was assessed immediately prior to each injection using the spectrophotometric assay at 240 nm on SmartSpec instrument (Bio-Rad, USA). Hydrogen peroxide (100 uM) is stable for long time periods in a standard culturing medium without cells (Erol *et al.*, 2012), thus relatively fast hydrogen peroxide depletion in the cell culture represents an adequate stimulus for NFE2L2/AP-1 pathway activation. In our previous study we found that HeLa cells retain normal viability while having the NFE2L2/AP-1 pathway activated at hydrogen peroxide concentration in the medium of 400 uM when treated for 24 h (Belanova *et al.*, 2017). A 24-h incubation period was used, based on the observation that full-scale activation of several NFE2L2/AP-1 pathway targets is achieved and stabilized by 24 h, and this treatment period is used in numerous related studies (Marrot *et al.*, 2008). For routine cell viability screening, we used a trypan blue exclusion assay. There were 8 samples in each group.

### RNA isolation

The RNA isolation procedures and cDNA synthesis set-up were performed in a laminar flow cabinet decontaminated with RNaseZap anti-RNase reagent (Sigma, USA). RNA was isolated using the Qiazol lysis reagent (Qiagen, The Netherlands) according to the standard phenol-lysis modification of the acidic phenolic method (Chomczynski and Sacchi, 2006). Phase separation was achieved by adding of 1-bromo-3-chloropropane (Sigma, USA). RNA was precipitated with isopropanol (Vekton CJSC, Russia) and then twice washed with 75% purified ethanol. The RNA pellet was dissolved in DEPC-treated water (Syntol LLC, Russia), heated, mixed and aliquoted for checking of RNA integrity and purity, genomic DNA contamination control, and reverse transcription.

RNA integrity was assessed using non-denaturing 1% agarose gel electrophoresis (Amresco, USA; Lytech LLC, Russia; Helikon LLC, Russia; DNA-technology LLC, Russia). The gel was stained with ethidium bromide (Lytech LLC, Russia) and bands visualized on a GelDoc XR system (Bio-Rad, USA). All samples had bright distinct rRNA bands, a mixed 5.8S/5S/tRNA band, a normal mRNA smear, and no visual signs of degradation (Supplementary Figure S1). The spectrophotometric assays were performed using the Nanodrop-1000 instrument (Thermo Fisher Scientific, USA). All samples had a A260/280 ratio ≥ 1.8, ranging between 1.83 and 1.97, as well as no signs of significant ethanol carry-over.

Genomic DNA or other template/primer contamination was checked as a standard reverse transcription reaction mixture without the enzyme run in qPCR reactions with the primers to the chosen regions of the mRNAs. The details on the qPCR protocol are provided below. No samples had a qPCR curve reaching the quantification threshold before the 40^th^ cycle.

RNA was reverse-transcribed using a kit from Syntol LLC (Russia) according to the manufacturer's protocol with an oligo(dT) primer. The reaction was run for 1 h at 39 °C, and then the enzyme was inactivated by a 5 min incubation at 92 °C. The cDNA was stored at −20 °C.

### Primer design and synthesis, and quantitative PCR

We designed primers for *GPX2* (one protein-coding transcript and two NMD-transcripts), *NQO1* (four transcript variants), *SQSTM1* (three transcript variants), *TBP* (reference gene) and *POLR2C* (reference gene) according to the standard selection procedure using the most recent GenBank reference RNA sequences (NCBI Gene), Oligo 7 software for primer selection, OligoCalc and IDT Oligo Analyzer for the melting temperature analysis consistency test, and NCBI primer BLAST for the *in silico* specificity test (against the refseqRNA database). The primers were selected so as to span exon-exon junctions. The *GPX2* NMD (nonsense-mediated decay) transcript variant 2 primers required a single LNA modification to normalize the thermodynamic properties of the pair. The primer sequences are given in Supplementary Table S1. Oligonucleotides were synthesized by Syntol LLC (Russia) and dissolved in DEPC-treated water (Syntol, Russia).

Quantitative PCR (qPCR; the SYBRGreen type; FAM channel detection) was performed using hot-start EvaGreen qPCR kits from Syntrol LLC (Russia) or OneTaq Hot Start DNA Polymerase with GC-buffer (New England Biolabs, USA) on a CFX96 instrument (Bio-Rad, USA). The latter was only used for the *SQSTM1* transcript variant 1 (tv1) cDNA, as this amplicon required higher annealing temperatures than the other targets. In this case, the *SQSTM1* tv1 reaction mixes were always run with both *POLR2C* and *TBP*, with a higher melting temperature set for the *SQSTM1* transcript variant 1 wells using the gradient function. In these settings, *POLR2C* and *TBP* had 0.3 °C difference in the annealing temperatures, which was negligible as it was seen from the PCR optimization set-ups (data not shown). The reaction parameters were as follows: 94 °C for 5 min (the polymerase activation step); 35 cycles (40 cycles in the negative control reaction set-ups) of 94 °C for 15 s, 57.5 °C (60 °C for SQSTM1 tv1 and thermal gradient in the amplification specificity check set-ups) for 20 s, 70 °C for 30 s, followed by a melting analysis (0.5 °C increment from 50 to 95 °C; 20 s per cycle).

The reaction optimization and characterization included gradient PCR for determining the effective annealing temperature and a primer efficiency test performed in 4-step two-fold serial dilutions for each primer pair. All reactions had efficiency within an acceptable range. Reaction specificity was controlled using the melting curve analysis and 1.5% agarose gel electrophoresis for each primer pair in several repeats. No abnormal products were detected.

### RNA folding analysis

RNA folding predictions were performed using the RNAfold web tool (Vienna RNA servers). MFE structures are presented in Results.

### Data analysis

qPCR data were analyzed using CFX96 system software (Bio-Rad, USA). Quantification threshold was set at the level of the early logarithmic phase of the qPCR curves, and it was the same in all reactions.

The Ct data were normalized using the standard deltaCt algorithm expressed by the formula R=(2*E)^(-deltaCt), where R is the ratio of expression of the target and reference (or regulating - *NFE2L2*) genes; E is the target gene reaction efficiency expressed as a proportion; deltaCt is the arithmetic diff between the target and reference genes. Data for the target genes were normalized to the both reference genes independently and to their geometric mean.

Statistical analysis was performed using SPSS22 software (IBM, USA). All data were tested for normality (Kolmogorov-Smirnov test). Parametric (ANOVA, Pearson's correlation) and non-parametric (Mann-Whitney, Spearman's correlation, and the Fisher r-to-z transformation) tests were employed in accordance with the normality testing results. All data were normally distributed and thus tested with both parametric and non-parametric criteria so as to assess the statistical consistency. All calculations were performed with appropriate adjustments for small groups in order to reduce a false-positive hypothesis rejection rate. Expression data (in relative units, r.u.) are given as mean (m) ± SD. First-type error was considered acceptable when below 0.05.

## Results

### Properties testing of reference genes

Reference gene selection is a major challenge in gene expression studies. In the present study we used two reference genes, *TBP* and *POLR2C*, that had been previously proven in our lab to be adequate for experimental model of hydrogen peroxide treatment of HeLa cells (Belanova *et al.*, 2017). In the present study, we re-evaluated these data. The results are given in Table 1. As seen from the data, *TBP* and *POLR2C* have highly congruent expression in the control and treatment groups.

**Table 1:** Results of correlation analysis of *TBP* and *POLR2C* expression.

Correlation analysis type	Control		Hydrogen peroxide, 400 uM, 24 h		Differences between the groups
	Correlation coefficient	p-level	Correlation coefficient	p-level	
Parametric testing	0.857	*0.029*	0.843	*0.009*	Non-significant
Non-parametric testing	0.886	*0.019*	0.929	*0.001*	Non-significant

### GPX2, NQO1 and SQSTM1 transcript variants folding

**Figure 1:**
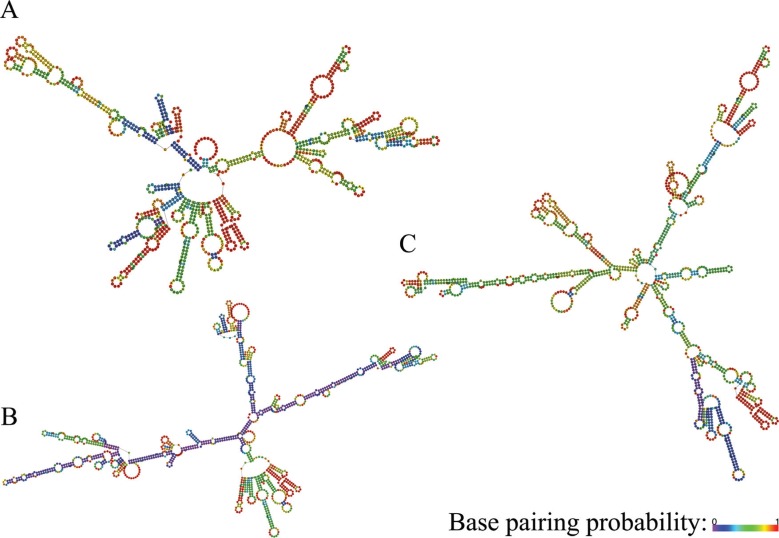
Folding patterns of *GPX2* transcript variants. A - transcript variant 1 (protein-coding); B - transcript variant 2 (NMD); C - transcript variant 3 (NMD). Color-scale represents base pairing probabilities: violet and red correspond to 0 and 1 probabilities, respectively.

**Figure 2:**
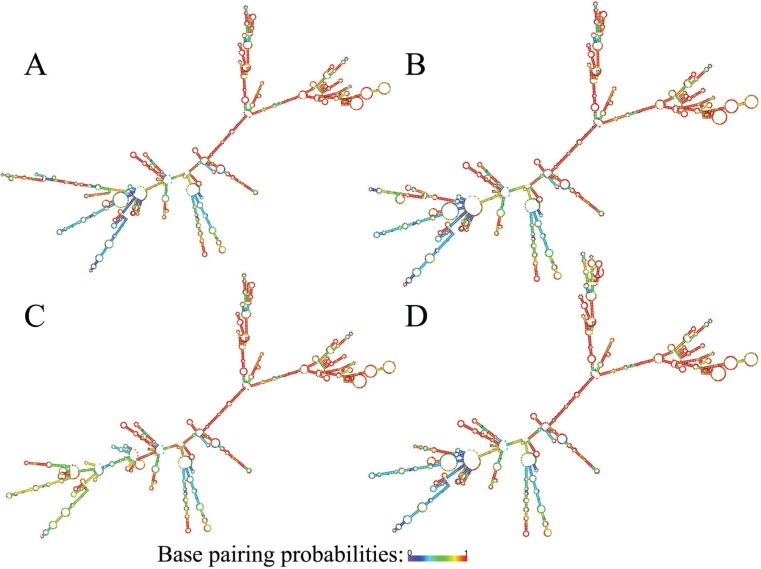
Folding patterns of *NQO1* transcript variants. A - transcript variant 1; B - transcript variant 2; C - transcript variant 3; D - transcript variant 4. Color-scale represents base pairing probabilities: violet and red correspond to 0 and 1 probabilities, respectively.

**Figure 3:**
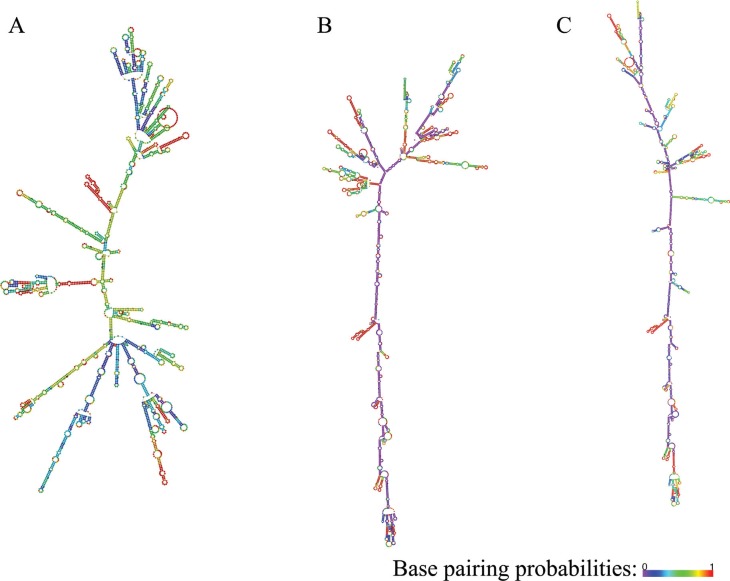
Folding patterns of *SQSTM1* transcript variants. A - transcript variant 1; B - transcript variant 2; C - transcript variant 3. Color-scale represents base pairing probabilities: violet and red correspond to 0 and 1 probabilities, respectively.

Predicted folding patterns of the *GPX2, NQO1* and *SQSTM1* transcript variants are shown in Figure 1, Figure 2 and Figure 3, respectively. As seen from the figures, transcript variants of the three genes have significantly different folding.

### Expression of the *GPX2*, *NQO1* and *SQSTM1* transcript variants in hydrogen peroxide-treated HeLa cells

**Figure 4:**
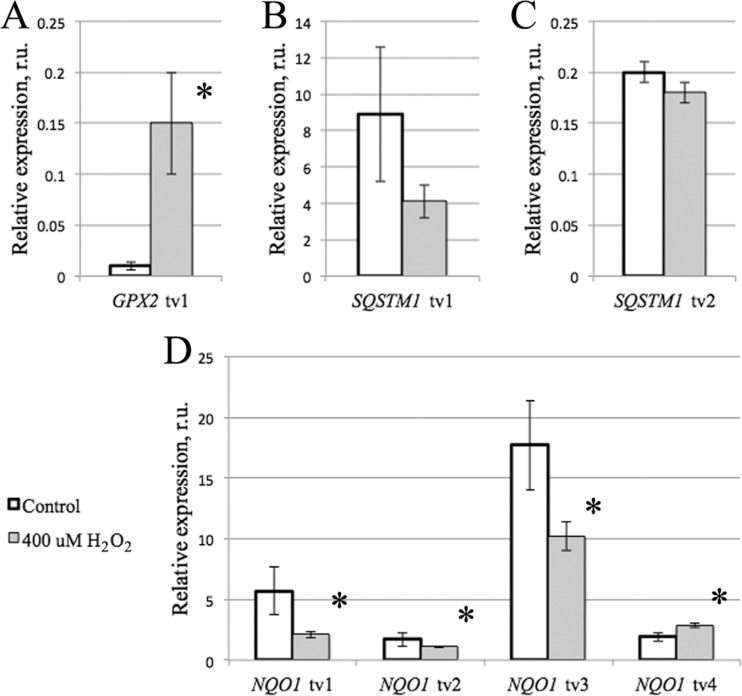
Character of expression of *GPX2* (A), *SQSTM1* (B, C) and *NQO1* (D) transcript variants. * - p < 0.05 - differences are significant in at least one of the normalization methods.

The expression analysis results are given in Table 2, and representative graphs are provided in Figure 4. As seen from Table 2, in the settings tested, the *GPX2* NMD-transcripts had no detectable expression. At the same time, *GPX2* transcript variant 1 was pronouncedly induced by 400 uM hydrogen peroxide treatment (Figure 4A). The results were consistent when statistically tested using both parametric and non-parametric methods, and were similar disregarding the normalization method.

**Table 2:** Expression of the *GPX2, NQO1, SQSTM1* transcript variants in control cells and in HeLa cells treated with 400 uM of hydrogen peroxide.

Group	Control			Hydrogen peroxide, 400, uM 24 h		
Normalization:	*TBP*	*POLR2C*	Double normalization	*TBP*	*POLR2C*	Double normalization
*GPX2* tv1	0.007 ± 0.004	0.01 ± 0.004	0.01 ± 0.004	0.13 ± 0.04	0.16 ± 0.06	0.15 ± 0.05
p-level	-			*1: 0.019*	*1: 0.04*	*1: 0.028*
				*2: 0.001*	*2: 0.005*	*2: 0.005*
*GPX2* tv2		Expression undetectable			Expression undetectable	
p-level	-					
*GPX2* tv3		Expression undetectable			Expression undetectable	
p-level	-					
*NQO1* tv1	3.2 ± 0.99	5.7 ± 1.97	4.2 ± 1.38	1.8 ± 0.12	2.1 ± 0.26	1.9 ± 0.15
p-level		-		1: 0.14	*1: 0.054*	1: 0.082
			2: 0.28	*2: 0.08*	2: 0.081	
*NQO1* tv2	1.3 ± 0.45	2.2 ± 0.8	1.7 ± 0.6	1.03 ± 0.08	1.15 ± 0.1	1.1 ± 0.06
p-level		-		1: 0.47	1: 0.17	1: 0.27
				2: 1.0	2: 0.23	2: 0.85
*NQO1* tv3	9.7 ± 1.8	17.7 ± 3.7	12.9 ± 2.5	8.9 ± 0.5	10.2 ± 1.16	9.4 ± 0.65
p-level	-			1: 0.63	*1: 0.048*	1: 0.15
				2: 0.75	2: *0.06*	2: 0.34
*NQO1* tv4	1.50.33	2.6 ± 0.39	1.9 ± 0.33	2.80.2	3.1 ± 0.3	2.9 ± 0.2
p-level	-			*1: 0.06*	1: 0.305	*1: 0.019*
				*2: 0.02*	2: 0.181	*2: 0.043*
*SQSTM1* tv1	7.3 ± 2.9	11.4 ± 5.1	8.9 ± 3.7	4.1 ± 1.0	4.2 ± 0,8	4.1 ± 0,9
p-level	-			1: 0.27	1: 0.13	1: 0.18
				2: 1.0	2: 0.18	2: 0.66
*SQSTM1* tv2	0.2 ± 0.05	0.3 ± 0.06	0.2 ± 0.05	0.2 ± 0.02	0.2 ± 0.01	0.18 ± 0.01
p-level	-			1: 0.9	1: 0.13	1: 0.43
				2: 1.0	2: 0.41	2: 1.0
*SQSTM1* tv3		Expression undetectable			Expression undetectable	
p-level	-					

^1^ANOVA testing p-level; ^2^Mann-Whitney criterion testing p-level

*SQSTM1* transcript variants 1 and 2 (Figure 4B and 4C, respectively) did not show any changes in expression upon 400 uM hydrogen peroxide exposure. Transcript variant 3, however, was completely undetectable.

For *NQO1* (Figure 4D), we found clear evidence of differential regulation of expression of transcript variants. *NQO1* transcript variant 1 showed some decrease in expression in the treatment group, although only close to significance and not in the case of *TBP*-normalization. Transcript variant 2 did not show any significant expression differences between the groups. Transcript variant 3 demonstrated decreased expression in the hydrogen peroxide treatment group when normalized to *POLR2C*. In contrast, transcript variant 4 had higher expression in the treatment group with agreement of the *TBP* and double normalization methods.

## Discussion

Analyzing individual rather than total transcripts arising from a given gene is a far more effective approach when pathway signaling activity is considered. The pathway under analysis may control only one transcript or a set of transcripts of its target-gene, and the controlled transcripts may be regulated in opposite directions. Moreover, even when total resulting protein-coding capacity of an activated gene is in the focus, individual transcripts are still much more reliable than total RNA. The reason is that individually controlled transcripts also differ in their fate (storage, degradation or translation) and, in case of storage and translation, storage period and translation speed, respectively. Furthermore, biological significance of differential regulation of transcripts may be even greater, being linked to disease (Trombetta-Lima *et al.*, 2015) or miRNA or other processing control mechanisms (Laloo *et al.*, 2010). Due to these challenges in current cell biology, we aimed to test whether the NFE2L2/AP-1 pathway target genes *GPX2, NQO1* and *SQSTM1* exhibited individual transcript control when the cells were exposed to sub-lethal hydrogen peroxide treatment.

The transcripts of all these genes arise due to alternative splicing. Two of three transcripts of *GPX2* contain alternate internal exons rendering them subject to nonsense-mediated RNA decay (NCBI Gene). Three of four *NQO1* transcripts lack one or two in-frame exons, but still, all four RNAs are protein-coding (NCBI Gene). Two *SQSTM1* transcripts differ from the predominant transcript in 5'-UTR structure, yet, these transcripts also arise from alternative splicing (NCBI Gene). Although differential initiation of transcription has already been described for the NFE2L2/AP-1 pathway target gene *BACH1* (Jyrkkänen *et al.*, 2011), and this mechanism of transcript variants formation under control of a transcription factor is the one expected in the first place, alternative splicing is also often regulated by transcription factors. For example, an adjacent to the NFE2L2/AP-1 pathway, the SP1 pathway, controls a splicing factor SLU7, which, in turn, controls alternative splicing pattern(s) of the cell (Alberstein *et al.*, 2007). An example of a more specific splicing control is seen in the HIF1A pathway: HIF1A itself controls alternative splicing of its targets, including the well-known *PDK1* gene (Sena *et al.*, 2014). There are other examples of situations where transcription factors directly or indirectly control alternative splicing of their own targets and of other genes (Liu *et al.*, 2013). As known from these cases, transcription factors do so by regulating expression of splicing factors, or by direct effects on their target RNAs, or even by employing epigenetic machinery (Pan *et al.*, 2003; Luco *et al.*, 2010). Interestingly, the third case was actually described for an NFE2L2/AP-1 pathway component, the JDP2 transcription factor (Lerdrup *et al.*, 2005). JDP2 suppresses JUN activity and permits NFE2L2 activity (Tanigawa *et al.*, 2013), and also modulates alternative splicing of its targets via epigenetic mechanisms (Pan *et al.*, 2003; Luco *et al.*, 2010). Additionally, some genes do not have canonical TATA or CAAT boxes, and alternative genomic elements may serve for transcription initiation, bringing transcription factors and splicing control even closer (Malakooti *et al.*, 2001).

We found that, having a 15-fold increase in expression, *GPX2* was the most easily induced gene among those tested in our laboratory in this and our previous study [previously, we worked with *HMOX1*, *FTH1*, *CBR3*, *SESN2*, *GCLC*, *JUN* and *NFE2L2* using the same experimental model (Belanova *et al.*, 2017)]. In the present experimental settings, we could not detect expression of the *NMD* transcripts. However, under other conditions, the two *NMD* transcripts can be significantly up-regulated, and this expression character may not be similar to that of the protein-coding transcript.

*NQO1* was confirmed to have differential regulation of the transcripts in the settings tested. Transcripts 1 and 3 expression decreased (closely to significance in case of the transcript 1), while transcript 4 expression significantly increased. This is exactly the situation when total mRNA expression analysis would be insensitive to apparent, real changes in gene expression. Thus, all four transcripts differ in regulation, and one should establish the most responsive transcript for a study to be undertaken. One interesting question raised in the present study in this sense is what exactly are the transcripts controlled by NFE2L2 and AP-1. These two transcription factors may also control entirely or partially different transcripts.

*SQSTM1* also had a complex expression pattern. Transcript variant 3 was undetectable in the present settings. However, this transcript codes for a protein, and thus, its highly individual character of expression should be considered in further experiments. Transcript variants 1 and 2 did not demonstrate any differences in expression. However, *SQSTM1* was previously shown to be JUN-suppressed in the settings tested. Thus, the two transcripts may actually have pronounced differences in expression. We plan to test whether only one of the detected transcripts is negatively regulated by JUN. Nevertheless, it is obvious that analyzing individual, rather than total, transcripts is a preferred strategy for pathway activation studies, as well as for protein expression-based cellular responses tests.

From a structural point of view, all transcripts of all three studied genes have quite pronounced differences in folding. This highly plausibly implies differences in features of interactions with proteins determining RNA shuttling, storage, degradation and translation (Lin and Bundschuh, 2013; Ozretić *et al.*, 2015), rendering a given gene to serve different roles under different conditions and cellular contexts. In this sense, our results stress the need for further studies that will uncover functional insights into biological roles of differential expression of the *GPX2*, *NQO1* and *SQSTM1* transcript variants.

There were several limitations of our study that we would like to outline and discuss. Firstly, as this was the first study for the set of genes we chose and one of only few studies on differential regulation of transcripts expression in general, we used only one fundamental model of activation of the NFE2L2/AP-1 pathway. We plan to study other stimuli in the future and anticipate that there will be slight differences in responses of the transcripts due to changes in the general cellular context. Secondly, we did not study the biological roles of the differential expression of transcript variants. As for the current study, we did not aim or plan to do it, since we could not predict the results of the study. This major and extremely complex problem, which concerns the biological significance of individual transcript variant control, requires a separate thorough investigation. However, we feel that these limitations do not compromise our findings, which will definitely be helpful for future studies in molecular biology of the cell and molecular medicine.

To summarize the results of the study, all three genes tested, *GPX2, NQO1* and *SQSTM1*, were characterized by individual control of transcript variants expression in HeLa cells treated with 400 uM hydrogen peroxide. These features of the genes should be accounted for in experiments designed for the NFE2L2/AP-1 pathway activation-based studies, as well as in related and similar projects, as some transcripts of these genes have opposite regulation. These features of the studied genes, along with highly distinct folding patterns of their transcripts, also suggest significant differences in the biological roles of the transcript variants.

## Supplementary material

The following online material is available for this article:

Figure S1 - RNA integrity testing results.

Table S1 - Primer sequences used in the study.
