# Supplementary material for: Individual expression features of GPX2, NQO1 and SQSTM1 transcript variants induced by hydrogen peroxide treatment in HeLa cells
Source: Genet Mol Biol. 2017 May 29;40(2):515–24. doi: 10.1590/1678-4685-GMB-2016-0005 (PMC5488449; doi:10.1590/1678-4685-GMB-2016-0005)
Supplement: Supplementary file 1 [file 1415-4757-gmb-1678-4685-GMB-2016-0005-Suppl01.pdf]

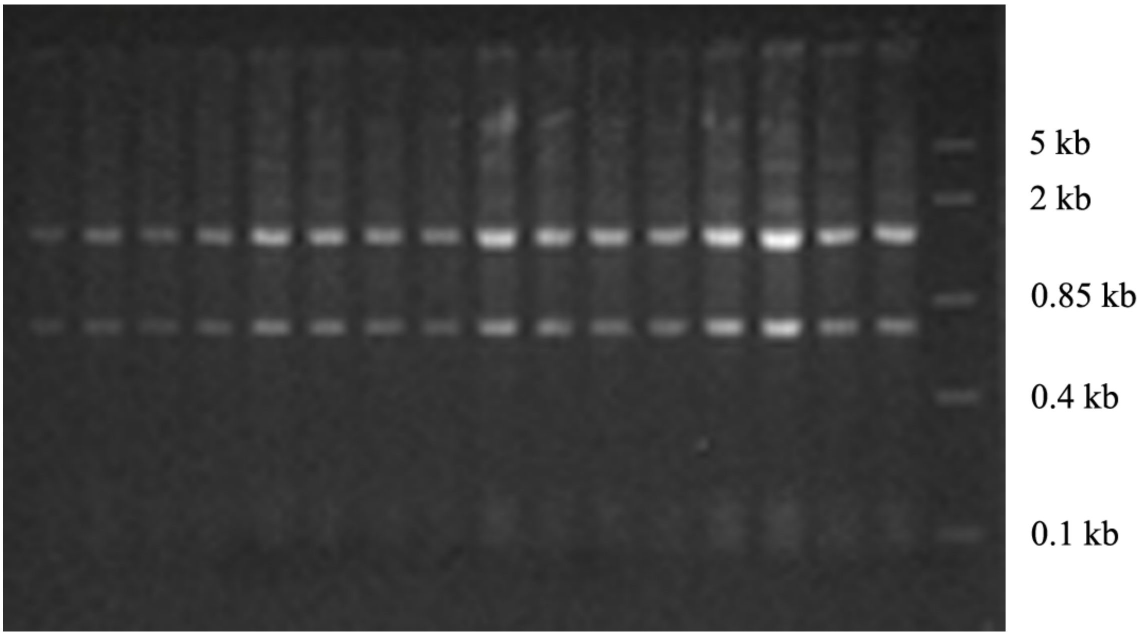

Figure S1 - RNA integrity testing results. Gel electrophoresis settings: 1% agarose TAE-buffered gel, 5.5 V/cm, 1 h, ethidium bromide staining.
