# Supplementary material for: Individual expression features of GPX2, NQO1 and SQSTM1 transcript variants induced by hydrogen peroxide treatment in HeLa cells
Source: Genet Mol Biol. 2017 May 29;40(2):515–24. doi: 10.1590/1678-4685-GMB-2016-0005 (PMC5488449; doi:10.1590/1678-4685-GMB-2016-0005)
Supplement: Supplementary file 2 [file 1415-4757-gmb-1678-4685-GMB-2016-0005-Suppl02.pdf]

Table S1 - Primer sequences used in the study

Target	Forward, 5’-3’	Reverse, 5’-3’	Notes
GPX2 tv 1	ATTTGGACATCAGGAGAACTGT	CTTCAGGTAGGCGAAGACA	
GPX2 tv 2	ATTTGGAC+ATCAGAACTGCTCA	GCTGCTCTTCAAGATTTAGCACT	“+” denotes an LNA-modification of the preceding nucleotide
GPX2 tv 3	ACCATCTCTTCAGCCTAGCAAG	TCTGACAGTTCTCCTTAGCCTC	
NQO1 tv 1	CAAAGGACCCTTCCGGAGT	CCCTTGCAGAGAGTACATGG	
NQO1 tv 2	TGCTTACACTTACGCTGCCAT	TGCAGAATGCCACTCCGGAA	
NQO1 tv 3	CCTTGTGATATTCCAGAGTAAGAAG	GTGCCCAATGCTATATGTCAGT	
NQO1 tv 4	TTGTGATATTCCAGAGTGGCATT	ACAGTGGTGTCTCATCCCAA	
SQSTM1 tv 1	GCTATGGCGTCGCTCACCGTGAA	GTCCCCGTCCTCATCGCGGTA	This primer pair has increased Tm
SQSTM1 tv 2	GAGCCTCATCTCCTCGGTGTC	TCAGCTAAAAGCCAGGTTCTTGTG	
SQSTM1 tv 3	ATTTAAAGGGGCCGCAGCAC	AGTTCAGCTAAAAGCCAGGTTCT	
TBP	ATAATCCCAAGCGGTTTGCTG	AGAACTTAGCTGGAAAACCCAA	
POLR2C	CAGTACTCTCGGGACTGCACA	TCGCGTCTGGTCTTCATTGCAC	
